# Influence of WC Particle Size on the Mechanical Properties and Residual Stress of HVOF Thermally Sprayed WC–10Co–4Cr Coatings

**DOI:** 10.3390/ma15165537

**Published:** 2022-08-11

**Authors:** Kunyang Fan, Wenhuang Jiang, Vladimir Luzin, Taimin Gong, Wei Feng, Jesus Ruiz-Hervias, Pingping Yao

**Affiliations:** 1 School of Mechanical Engineering, Chengdu University, Chengdu 610106, China; 2 Sichuan Province Engineering Technology Research Center of Powder Metallurgy, Chengdu University, Chengdu 610106, China; 3 Australian Nuclear Science and Technology Organisation, Lucas Height, Sydney, NSW 2234, Australia; 4 School of Engineering, The University of Newcastle, Callaghan, NSW 2304, Australia; 5 State Key Laboratory of Powder Metallurgy, Central South University, Changsha 410083, China; 6 Materials Science Department, Universidad Politécnica de Madrid, Escuela Tecnica Superior de Ingenieros de Caminos, Canales y Puertos, C/Profesor Aranguren s/n, 28040 Madrid, Spain

**Keywords:** WC–10Co–4Cr, thermal spray, mechanical property, residual stress

## Abstract

Cermet coatings deposited using high-velocity oxy-fuel (HVOF) are widely used due to their excellent wear and corrosion resistance. The new agglomeration–rapid sintering method is an excellent candidate for the preparation of WC–Co–Cr feedstock powders. In this study, four different WC–10Co–4Cr feedstock powders containing WC particles of different sizes were prepared by the new agglomeration–rapid sintering method and deposited on steel substrates using the HVOF technique. The microstructures and mechanical properties of the coatings were investigated using scanning electron microscopy, X-ray diffraction, nanoindentation, and Vickers indentation. The through-thickness residual stress profiles of the coatings and substrate materials were determined using neutron diffraction. We found that the microstructures and mechanical properties of the coatings were strongly dependent on the WC particle size. Decarburization and anisotropic mechanical behaviors were exhibited in the coatings, especially in the nanostructured coating. The coatings containing nano- and medium-sized WC particles were dense and uniform, with a high Young’s modulus and hardness and the highest fracture toughness among the four coatings. As the WC particle size increased, the compressive stress in the coating increased considerably. Knowledge of these relationships enables the optimization of feedstock powder design to achieve superior mechanical performance of coatings in the future.

## 1. Introduction

Thermally sprayed WC–Co–Cr coatings have excellent wear performance and corrosion resistance and superior mechanical properties and are widely used in metallurgy, the petrochemical and energy industries, aerospace engineering, and other fields [1,2]. High-velocity oxy-fuel (HVOF) is the best thermal spray technique to obtain WC-based coatings, owing to the superior qualities of the deposited coatings (e.g., high density) and strong bonding between the substrate and the coating, as well as its environmentally friendly properties [3]. In the HVOF coating process, the preparation of the spraying feedstock powders (e.g., agglomeration sintering method) is time- and energy-consuming, resulting in high costs and low production efficiency, which limits its engineering application. To solve these problems, Zhou et al. [4] reported a new agglomeration–rapid sintering method. The spray granulation powders were sintered in a vertical ultra-high-temperature sintering furnace in the free-falling mode. The combination of the starting particles in the spray granulation powders (e.g., tungsten carbide powder and cobalt) was changed from the initial mechanical combination to metallurgical combination in a very short time (approximately 1–5 s), which simplified the process and improved the production efficiency. The aggregation between powders, which usually occurs in the traditional agglomeration sintering process, could be effectively avoided; thus, it can guarantee the good fluidity of the feedstock powders during spraying. Meanwhile, it was ensured that the obtained WC–Co–Cr spraying powders had a certain structural strength, which is beneficial for obtaining a dense coating during subsequent spraying. 

Previous research has shown that the WC particle size can significantly affect the WC–Co (or WC–Co–Cr) coating characteristics, such as the phase, microstructure, mechanical properties, and abrasive wear resistance [5,6,7]. However, different results, even opposite ones, have been reported in other studies. For example, Dent [8] and E. Sánchez et al. [9,10] found that with a decrease in the WC particle size, the oxidation and decarburization of WC particles are more pronounced during deposition, the hardness of the coating increases, and the toughness and wear resistance decrease. Other studies [11,12,13,14] observed that the hardness and fracture toughness of coatings with nano-sized WC particles increased significantly, leading to a significant improvement in wear resistance. Several studies found that the bimodal WC–Co coating with combined micro- and nano-sized WC particles exhibited enhanced hardness and wear resistance compared to the conventional WC–Co coatings [15,16]. However, Gong et al. [5] reported that the WC–10Co–4Cr coating containing medium-sized (~1.2 μm) WC particles exhibited the highest hardness and the best abrasive wear resistance over other coatings containing fine or bimodal WC particles. These inconsistent results bring unreliability for the design and quality control of coatings in application. To guarantee the quality of feedstock powders prepared by the new agglomeration–rapid sintering, and thus improve the properties of the sprayed coatings, it is important to further investigate the relationship between the WC size and the microstructures and mechanical properties of WC–Co–Cr coatings. 

In addition, to bear thermo-mechanical loads with no failure, the residual stress of the WC–Co–Cr coating is a critical issue in the overall performance of the coated components. Residual stresses generated during the HVOF spraying process are a complex superposition of the thermal stresses due to the thermal expansion mismatch between the coating and substrate and the deposition stress associated with the deposition process [17]. Since the residual stress has a significant influence on the mechanical properties of the components and their overall performance, it is desirable to clearly understand the formation mechanism and tailor it for specific practical applications. For example, in wear applications, compressive residual stresses can strengthen the coating and improve its wear resistance [18]. Residual stresses can be controlled by the choice of suitable sprayed materials and spraying processes. Previously published studies [18,19] were mainly concerned with the relationship between residual stresses and HVOF spraying parameters (i.e., temperature, feed rate, and particle velocity), whereas the influence of the starting powders on residual stresses has rarely been reported. Since the spraying parameters of different studies are inconsistent, forming conclusions about the WC grain size effect is practically impossible. It is highly interesting to determine the influence of WC particle size on the development of residual stresses in the HVOF thermally sprayed coatings. Knowledge of the residual stresses depending on the starting powder parameters is essential for the quality control of HVOF-sprayed WC–Co–Cr coatings. Therefore, the precise determination and understanding of the nature and magnitude of the residual stresses between the coating and the substrate is required.

There are many techniques for residual stress measurement, including X-ray diffraction (XRD), the curvature method, the hole drilling method, and computational modeling [20]. Considering the coating specimen geometry limitation (e.g., limited thickness) and testing precision, neutron diffraction has been suggested as a suitable method for stress measurements in WC–Co–Cr coatings [21,22]. Neutron diffraction is a superior method for internal strain measurement with high resolution and deep penetration of neutrons into materials [23], which facilitates obtaining reliable depth-resolved stress data of both the coatings and substrates. 

In this study, WC–10Co–4Cr feedstock powders containing different WC particle sizes were prepared by a new agglomeration–rapid sintering method, and the coatings were deposited using the HVOF technique. The microstructures and phase compositions of the feedstock powders and coatings were investigated. The mechanical properties, such as the hardness, Young’s modulus, and fracture toughness, of the coatings were studied using nanoindentation and Vickers indentation. The through-thickness residual stress profiles of the coating and substrate materials were determined by means of the neutron diffraction technique. This study aimed to establish the relationship between the WC size, WC–Co–Cr feedstock powders, microstructures, mechanical properties, and corresponding residual stresses of HVOF sprayed WC–Co–Cr coatings.

## 2. Materials and Methods

### 2.1. Material Processing and Basic Characterization

**Figure 1:**
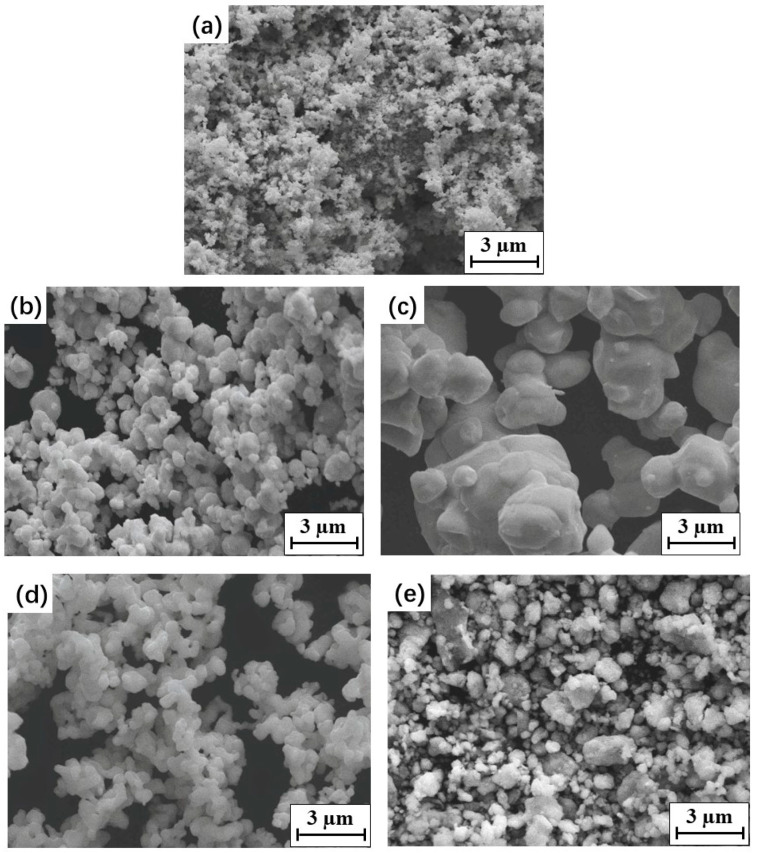
Morphology of the WC, Co, and Cr starting powders: (**a**) nano WC, (**b**) medium WC, (**c**) coarse WC, (**d**) Co, (**e**) Cr.

The materials used in this study were HVOF thermally sprayed WC–10Co–4Cr coatings. WC–10Co–4Cr feedstock powders were prepared by the new agglomeration–rapid sintering method [4] with primary WC, Co, and Cr particles. WC particles of three different sizes were used as the raw materials: nano (100–300 nm), medium (0.8–1.5 µm), and coarse (4–5 µm). The morphology of the starting powders is shown in Figure 1. Four different mixtures of spraying feedstock powders with different WC particle sizes were prepared (Table 1). The N and M feedstock powders contained nano- and medium-sized WC particles, respectively. The B1 bimodal feedstock powders were composed of nano- and medium-sized WC particles, and the B2 bimodal feedstock powders were mixed with medium-sized and coarse WC particles.

The detailed process of the new agglomeration–rapid sintering method used in the present work is described as follows. The starting powders (WC, Co, and Cr powders) were mixed according to the compositions listed in Table 1. Stable slurries were prepared by mixing the starting powders in deionized water, using 3.0 wt.% polyethylene glycol 6000 as a binder and 1.0 wt.% polyethylene glycol 2000 as a dispersant. The slurries were ball-milled for 10 h using an alumina zirconia jar and balls. The prepared slurry was spray-granulated using a PGZ-15KL centrifugal spray-drying tower (ACME, Changsha, China). After degreasing, spherical agglomerates were quickly sintered in a GWQHL-70-03 vertical ultra-high-temperature instantaneous melting spheroidizing furnace (FULLAD, Zhuzhou, China). The sintering was performed by the continuous free-fall feeding of the powders through the heating and cooling zones in the furnace. The sintering temperature was 1250 °C, with a sintering time of 1–5 s, and the protective atmosphere was nitrogen. Finally, the sintered powders were sieved to obtain feedstock powders with suitable particle sizes (15–50 μm) for the HVOF thermal spraying. 

Steel plates (ASTM1045) with dimensions of 80 × 40 × 6 mm were used as the substrates. Prior to spraying, the steel substrates were ultrasonically cleaned with acetone and grit-blasted using 250 μm alumina abrasive particles under 0.4 MPa pressure for 30 s to obtain a surface roughness of Ra = 5 μm. The coatings with a thickness of approximately 250 μm were deposited using a Tafa JP5000 HVOF spraying system (Praxair, Danbury, CT, USA). A detailed description of the system is given elsewhere [24]. In order to focus on the WC grain size effect without introducing other factors, the spraying parameters for the HVOF process were kept constant for each coating, as listed in Table 2. 

After the deposition, small test pieces were cut from the resulting samples, and the cross-sectional coating samples were ground and polished using diamond abrasives. Metallurgical samples of the spraying feedstock powder were prepared by hot mounting, grinding, and polishing. The morphologies and cross-sections of powder and coating were studied by scanning electron microscopy (SEM). The elemental composition of the coatings was investigated using an energy-dispersive X-ray spectrometer (EDS) attached to the SEM. Based on the SEM images of the cross-sectional coatings, the average grain sizes of WC and the mean free path of the binder in the coating were determined using the linear intercept method [13], and the porosity of the coatings was measured by the grayscale method using image analysis software (Leica Q500MC, Wetzlar, Germany). The phase identification of the feedstock powders and coatings was performed by X-ray diffraction (XRD) using Cu Kα radiation at room temperature over a 2θ angle range of 10–80°, with a step size of 0.03° and a counting time of 1.0 s at each step (Bruker D8, Bruker Corporation, Billerica, MA, USA). A quantitative phase analysis was performed using the Rietveld pattern-fitting method. 

### 2.2. Nanoindentation Testing

The nanohardness (H) and Young’s modulus (E) of each coating were measured by nanoindentation tests using a Nanoindenter XP instrument (Nanoinstruments Innovation Center, MTS systems, Oak Ridge, TN, USA). A Berkovich diamond indenter with a 50 nm tip radius was used. The tests were performed using a continuous stiffness measurement (CSM) method [25]. Prior to the nanoindentation test, the system was calibrated using a fused silica standard. An array of 25 indentations in a 5 × 5 matrix with 50 µm spacing was made on the coating surface of each sample, with a maximum penetration depth of 1000 nm. To determine the mechanical properties through the thickness of the coating, an array of 30 indentations in a 3 × 10 matrix with 25 µm spacing was made on the coating’s cross-section of each sample. Based on the obtained indentation curves, the H and E values were calculated as a function of the penetration depth using the method of Oliver and Pharr [26].

### 2.3. Vickers Indentation Testing

In addition to the nanoindentation test, the microhardness ($H_{V}$) of the coatings was determined via Vickers indentation on a polished coating surface with a load of 300 g for a dwell time of 15 s. 

The indentation fracture toughness ($K_{IC}$) of the coatings was measured by the indentation method with a load of 5 kg and a dwell time of 15 s. Based on the measurements of the Vickers indentation crack lengths and imprint area using optical microscopy, the types of crack system were identified as median (half-penny) cracks, and the $K_{IC}$ values were calculated using the following equation [13,27,28].

(1) $$K_{IC}=0.079\frac{P}{a^{3/2}}\cdot \mathrm{lg}\frac{4.5a}{c}$$

where P is the applied load (N), a is the half-diagonal length of the indented section (m), and c is the crack length from the center of the indent to the crack tip (m). To obtain the average values and standard deviations of the microhardness and fracture toughness, at least 10 indentation measurements were carried out for each coating. The SEM images of these indentations were analyzed to study the propagation of the corner cracks.

### 2.4. Residual Stress Measurements

Residual stress was measured using a constant-wavelength neutron strain scanner, KOWARI (ANSTO, Sydney, Australia) [29]. Samples of a 6-mm-thick steel substrate and 0.25-mm-thick WC–10Co–4Cr coating were used for residual stress investigations. Neutron diffraction scanning in the 0.25 mm coating thickness required the use of sub-millimeter spatial resolution, which inherently limited the neutron flux delivered to the sample. Considering the accuracy of stress measurements in the WC–10Co–4Cr coating, two methods were employed to determine the stress in the coating: (i) a direct measurement of the stress in the coating by neutron diffraction and (ii) an indirect approach [30], where the stress in the coating could be derived from the stress of the substrate by using stress balance conditions in the coating/substrate system. High accuracy and a sufficient number of scanning points in the substrate were essential to obtain reliable stress results for the coating using the second approach. 

Through-thickness measurements were conducted on the steel substrates with a scan step of 0.3 mm. The gauge volume of 0.4 × 0.4 × 18 mm^3^ was used for measurements in substrates, while 0.2 × 0.2 × 18 mm^3^ was used for measurements in coatings, considering the limited thickness of the coatings. The elongated gauge volume helps in maximizing the diffraction signal while maintaining the required spatial resolution. A near-to-90° geometry was used to measure both the coating and substrate materials by adjusting the wavelength through variations in the take-off angle of the Si(400) monochromator. A wavelength of 1.67 Å was used to measure the Fe(211) reflection in the substrate and 1.60 Å was employed to measure the WC(201) reflection in the coating. WC–10Co–4Cr feedstock powders were used as micro-stress-free reference samples for the evaluation of micro-stress. At each scanning point, measurements were conducted in the principal directions, normal and in-plane to the coating surface, in order to determine the normal and two in-plane strain components. The stresses were evaluated according to the measured strains in the principal directions following the balanced biaxial plane stress assumption [31]. The (hkl)-dependent elastic diffraction constants were used: S1 = −1.26 TPa^−1^ and ½S2 = 5.71 TPa^−1^ for Fe(211) reflection; S1 = −0.343 TPa^−1^ and ½S2 = 1.82 TPa^−1^ for WC(201). They were evaluated according to Kroner’s model using the IsoDEC software [32]. All samples were treated individually and investigated using the same procedure.

## 3. Results and Discussion

### 3.1. Microstructure and Phase Composition of the Feedstock Powders

**Figure 2:**
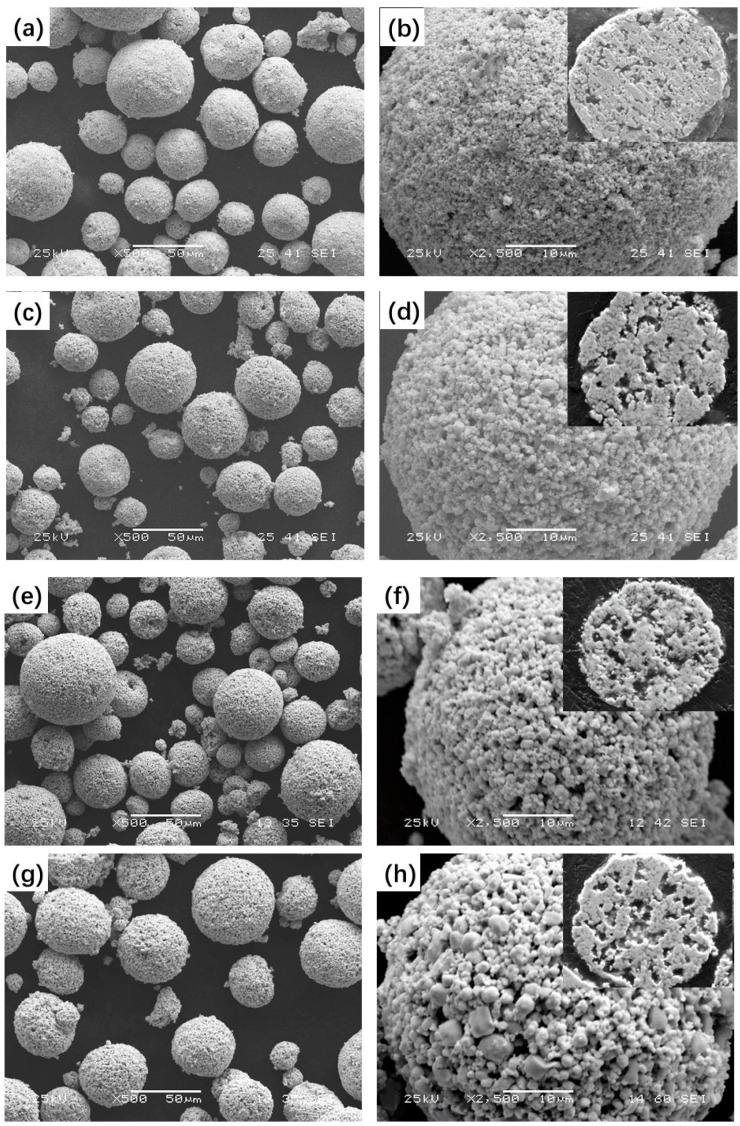
The morphologies and cross-sections of the four studied WC–10Co–4Cr spraying feedstock powders: (**a**,**b**) N; (**c**,**d**) B1; (**e**,**f**) M and (**g**,**h**) B2.

Figure 2 shows the typical morphologies and cross-sections of the four feedstock powders. The four types of feedstock powders had a near-spherical shape with a size of 15–50 μm, without obvious aggregation between powders. WC grains with various shapes and sizes were embedded in the metallic binder phase in each spherical feedstock powder. A porous structure was observed in both the surfaces and cross-sections of each feedstock powder. The porosity of the feedstock powders increased with the primary WC size. It was reported that an appropriate number of pores could benefit the absorption of high thermal energy during spraying [33]. 

**Figure 3:**
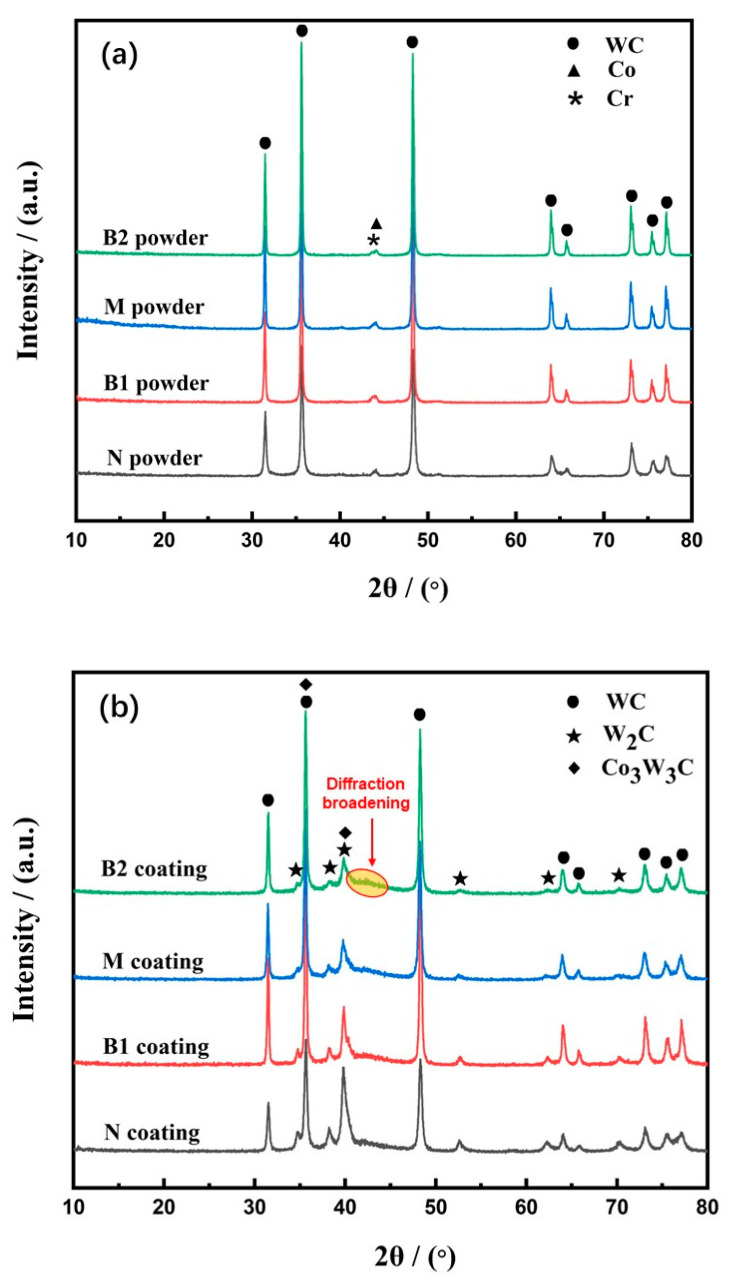
XRD patterns of the WC–10Co–4Cr feedstock powders (**a**) and the coatings (**b**).

The XRD patterns of the four sprayed feedstock powders, shown in Figure 3a, were similar, mainly consisting of WC and minor Co and Cr phases. Many studies have reported the generation of other phases (such as W_2_C, Co_3_W_3_C, and Co_6_W_6_C) during feedstock powder preparation because of the oxidation and dissolution of WC in the Co binder [9,13]. However, this would deteriorate the mechanical properties of the sprayed coatings [34]. No other phases were detected in the four studied spraying feedstock powders, indicating that no impurities or decarburization phenomena were introduced during their preparation. The new agglomeration–rapid sintering method, with a short sintering time and protective atmosphere, that was used to prepare feedstock powders was beneficial to control and prevent the generation of other phases; thus, it is favorable for guaranteeing the quality of the sprayed coatings.

### 3.2. Phase Composition of the Coatings

WC–10Co–4Cr coatings were obtained after HVOF spraying of the feedstock powders. The XRD patterns of the four WC–10Co–4Cr coatings are shown in Figure 3b. In addition to the main WC phase, new diffraction peaks compared with the feedstock powders are visible in all coatings. These peaks are ascribed to the W_2_C and Co_3_W_3_C phases. The lowest peak intensity of the WC phase and the highest peak intensity of the W_2_C and Co_3_W_3_C phases were observed in the diffraction patterns of the nanostructured N coatings. Quantitative phase analysis confirmed the highest content of W_2_C and Co_3_W_3_C phases in the N coating (Table 3). As the WC grain size increased, the content of W_2_C and Co_3_W_3_C phases in the coatings decreased.

The generation of the new W_2_C phase in the coatings can be attributed to the decarburization of the WC phase during the spraying process. The main reasons for decarburization during thermal spray deposition of the WC–Co (or WC–Co–Cr) coatings have been discussed in previous studies [7,35]: (i) thermal decomposition of WC due to direct contact with air during the spraying process, (ii) dissolution of carbide into the molten binder, and oxidation and decarburization when deposited, and (iii) physical rebounding of large carbide particles upon high-velocity impact. The Co_3_W_3_C phase was generated in relation to the dissolution of WC in the Co–Cr binder during spraying [17]. When the WC particle size decreased to nano-size, the surface-to-volume ratio—that is, the total surface energy—of the WC particles increased. As a result, the contact area between the WC and Co particles increased. This promoted serious decarburization and resulted in the favored generation of the W_2_C and Co_3_W_3_C phases. This might be the reason for the highest content of W_2_C and Co_3_W_3_C phases and the lowest retention of the WC phase in N coatings. Based on the measured W_2_C and Co_3_W_3_C phase content in each coating, decarburization could be restrained as the WC particle size increased to near micron size. 

The diffraction peaks of crystalline Co and Cr were not identified in the XRD patterns of the coatings, whereas a broad diffraction peak at 2θ = 40°–45° was observed, as shown in Figure 3b. It indicates the existence of an amorphous or nanocrystalline structure in the coating [2,36]. It is well known that W and C can dissolve in Co–Cr binders at high temperatures [7,37]. Owing to the instantaneous high-speed impact of high-temperature spraying particles on the cool substrate during HVOF spraying, the cooling rate is as high as 10^6^–10^7^ K/s [7], which makes it difficult for W and C to precipitate. Therefore, it promotes the formation of amorphous or nanocrystalline Co–Cr–W–C structures [35]. 

### 3.3. Microstructure of the Coatings

**Figure 4:**
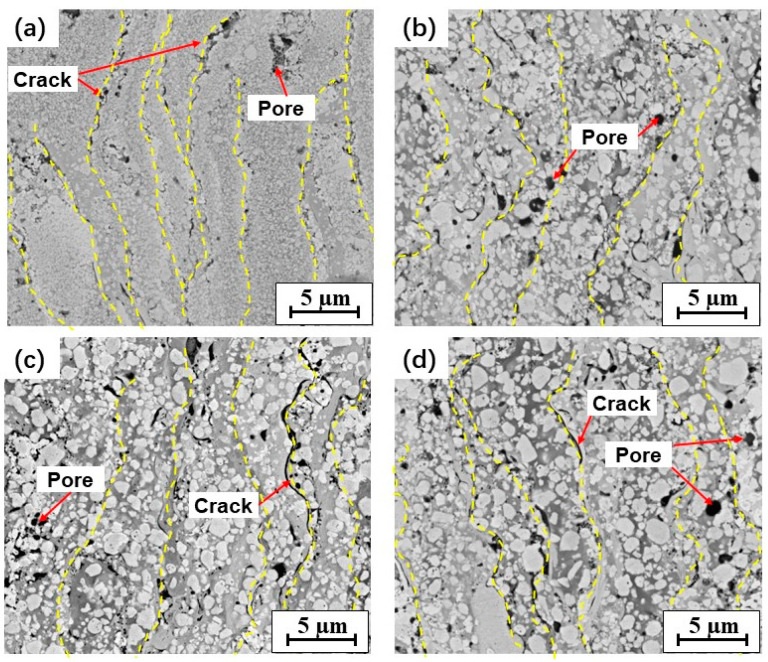
Cross-sectional BSE–SEM images of the studied WC–10Co–4Cr coatings: (**a**) N; (**b**) B1; (**c**) M; (**d**) B2.

Figure 4 shows the cross-sectional microstructures of the coatings. Dense coatings were obtained after HVOF spraying using four types of feedstock powders. The WC grains, in light gray color with rounded or polygonal shapes, were distributed almost evenly in the four coatings. The dark gray binder fully filled the WC particles. A lamellar-like structure was observed in the backscattered electron (BSE) SEM images of the four coatings (Figure 4) based on the clear differences in the brightness of the binder. The distinctly contrasting brightness levels indicated compositionally distinct regions in the BSE imaging mode, in which the brightness degree always increases with increasing atomic weight. According to the EDS results (Table 4), the binder is a Co-rich phase containing dissolved W, C, and Cr. A brighter color indicates a higher percentage of W in the binder, which results from the larger dissolution of WC into the liquid metallic binder during spraying. 

**Figure 5:**
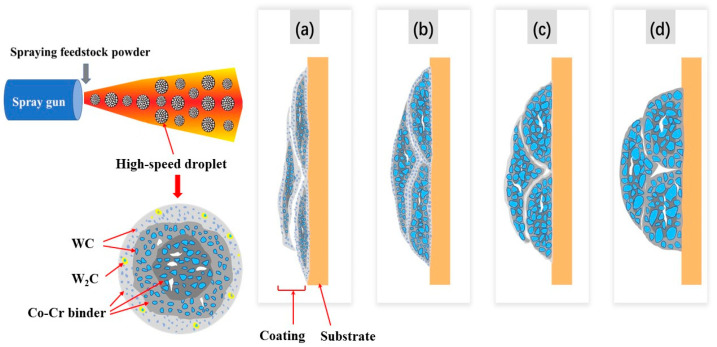
Schematic of the formation of the lamellar-like structures in HVOF sprayed WC–10Co–4Cr coatings with different WC particle sizes: (**a**) N; (**b**) B1; (**c**) M; (**d**) B2.

Such lamellar-like microstructural characteristics are highly related to the deposition process during spraying. As shown schematically in Figure 5, during the HVOF spraying process, the WC–10Co–4Cr feedstock powders were fed into the high-temperature flame flow and formed semi-molten droplets, in which WC was solid and the Co–Cr binder was molten. The semi-molten droplets spread over the substrate, and the fully molten binder phase moistened the WC particles and filled the gaps between the WC particles and the binder. As a result, a coating was generated through layer-by-layer deposition. Although the parameters of the HVOF process were relatively constant, the flight path of each particle may be slightly different in the flame flow. Therefore, different particles may have experienced slightly different flame flow temperatures and velocities. When the particles absorbed more heat or had a longer flight path, the W and C elements of WC were accelerated to dissolve in the melting Co–Cr binder. Furthermore, for each feedstock powder, the surface is in direct contact with the flame; thus, it experiences higher temperatures than the inner part. Partial overheating and more intense melting occurred on the droplet surfaces, while the inner part of the droplet was heated but did not sufficiently melt and remained predominantly in the solid state [17]. This led to greater dissolution of WC into the liquid binder on the outer surface of the droplets. After flat spread and deposition, it presents long strips with a brighter color around it. All these result in the lamellar-like structure in WC–10Co–4Cr coatings. 

Some pores and interlamellar cracks were observed in the WC–10Co–4Cr coatings. Fine globular pores may have been caused by outgassing reactions, e.g., CO was generated by the oxidation of WC and failed to escape from the coating during cooling. Coarse pores and cracks were present on the boundaries between the splat layers, which were related to the process of spreading and rapid solidification of droplets. This indicates weak cohesion between the splats in the coating. The porosities of the four coatings are presented in Table 3. Comparing the microstructures and porosities of the four WC–10Co–4Cr coatings, it was found that the coatings containing single-sized WC particles (i.e., N and M coatings) showed more pronounced lamellar-like structures and higher porosities than those containing bimodal-sized WC particles (i.e., B1 and B2 coatings). 

Most studies agree that the melting binder with nano-sized WC inclusion grains spreads more easily onto the substrate [13,17], resulting in thinner splat in the N coating. However, previous studies have also reported that the degree of droplet flattening is inversely proportional to the porosity of the coatings [7,38]. This is inconsistent with the results in the present work, as the N coating exhibited the most prominent lamellar-like structure and the highest porosity among all coatings. This inconsistency might be related to the effect of feedstock powder density. It is an important factor that determines the heating state of the droplets during HVOF spraying. The N feedstock powder with high density is not conducive to the high-temperature flame flow in the powder, which leads to uneven heating and decarburization in the droplet. Partial overheating and stronger decarburization occurred at the surface than in the inner part of the feedstock powders. This resulted in an uneven composition in the droplet, which makes it difficult to reach ideal wettability when the semi-molten droplet overlaps with the previously deposited coating. This is detrimental to the mutual filling and elimination of defects between the deposited droplets, leading to weak bonding, such as pores and cracks between the splats (Figure 4a). Among all feedstock powders, such uneven decarburization is the most pronounced in the N powder, resulting in the highest porosity in the N coating. This implies that the heating uniformity of the feedstock powder has a significant influence on the wettability and bonding between droplets, thereby affecting the microstructure and porosity of the thermally sprayed coatings. 

As the WC size increased to the micron size, the low-density spraying powder could be heated more uniformly, and the decarburization was significantly reduced. This is conducive to wettability and bonding between the closed droplets, which is beneficial for obtaining a dense coating. However, the increase in WC size hindered sufficient deformation of the droplet during deposition, which is not beneficial for the droplet flattening behavior. The feedstock powders containing bimodal-sized WC particles, particularly in the B1 coating containing nano- and medium-sized WC particles, can offer a compromise between the two effects described above to obtain a relatively dense coating. This led to the lowest porosity of the B1 coating.

**Figure 6:**
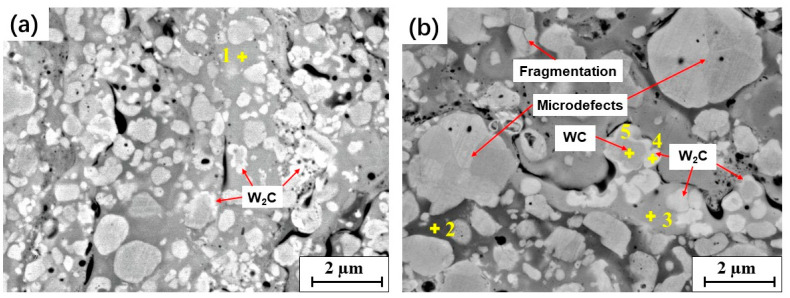
W_2_C formation and fragmentation of the WC phases in the coatings: (**a**) B1; (**b**) B2.

In the BSE–SEM images at high magnification, WC particles with a surrounding white rim were also observed in the coatings, as shown in Figure 6. A similar phenomenon was reported in previous studies [5,17]. According to the EDS analysis (Table 4), the white rim can be ascribed to the W_2_C phase, which formed due to the decarburization of WC during spraying. As mentioned above, the WC particles were wetted by the melting binder when deposited, leading to the oxidization of C. Thus, the W_2_C phase was easily formed at the interface between the WC particles and melting binder, occurring as a white rim surrounding the WC particles on the SEM images. This phenomenon was common in the surface region of each splat in the coating, especially in coatings that contained fine WC particles.

In addition, some microdefects and fragmentations were observed in the coarse WC particles of the B2 coatings, as shown in Figure 6b. The coarse WC grains in the spray feedstock powders led to a greater impact on the coating, and, therefore, a higher counteracting force on the WC grains. This resulted in an increase in microdefects, such as dislocations and fragmentation, in WC particles [39]. 

### 3.4. Young’s Modulus and Hardness

**Figure 7:**
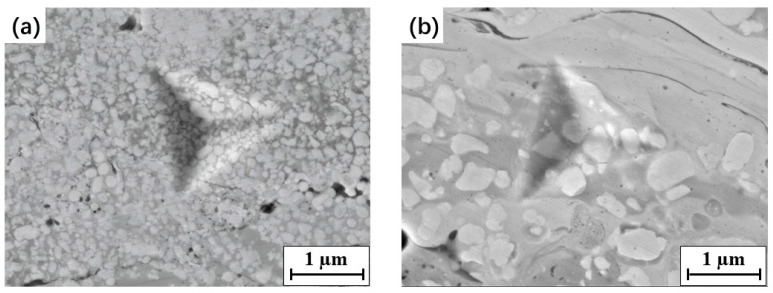
The typical SEM micrographs of nanoindentation imprint on the coatings: (**a**) N; (**b**) M.

Nanoindentation tests were carried out on both the surface and cross-section of the coatings to measure the nanohardness (H) and Young’s modulus (E) in different directions. A typical SEM micrograph of a 1000 nm penetration depth imprint on the coating surface is shown in Figure 7. It can be observed that these indentations are large enough to include a representative portion of the microstructures and, consequently, to test the mechanical behavior of the coatings. 

**Figure 8:**
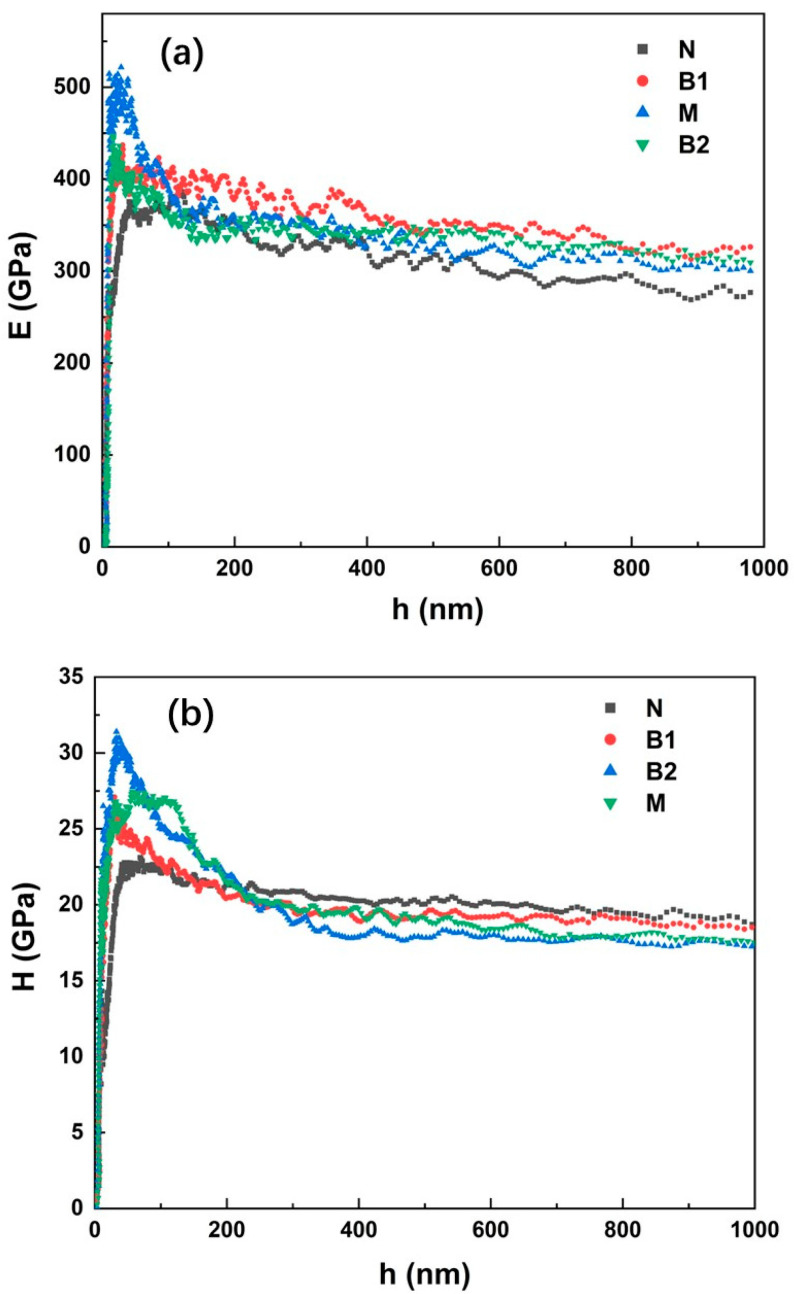
Young’s modulus (E) and nanohardness (H) vs. penetration depth (h) curves on the coating surfaces. (**a**) Young’s modulus; (**b**) nanohardness.

The represented evolution of E and H as a function of the penetration depth (h) for the four coating materials is presented in Figure 8a,b, respectively. Because of the implicit experimental variability of the factors, such as tip–sample interactions, sample roughness, and especially tip rounding, E and H values are severely affected in the initial penetration depth regime [40]. As the indenter tip was inserted deeper than 300 nm, reliable values of E and H were determined for each coating sample, which were independent of the penetration depth. The average E and H values measured on the coating surfaces and cross-sections are listed in Table 5. Higher values of E and H on the coating surfaces than in the corresponding cross-sections could be observed for all coatings, indicating an anisotropic mechanical behavior. Such mechanical anisotropy is most pronounced in the nanostructured N coating and is slightly reduced as the WC particle size increases. Considering the deposition process in the thermal spraying process, the droplets in the N coating were spread more sufficiently, resulting in a more pronounced lamellar-like structure and remarkable anisotropy in the mechanical properties compared to the other coatings. 

Among all four WC–10Co–4Cr coatings, the highest nanohardness value was observed for the N coating, while its Young’s modulus was the lowest. In contrast, the B1 coating exhibited the highest Young’s modulus. These values can be explained by observing the microstructures and phase compositions of the coatings. Previous studies [41,42] have shown that the Young’s modulus and hardness of WC–Co materials (or WC–Co–Cr) strongly depend on various parameters, such as the grain size, volume fraction and contiguity of WC particles, mean free path of the binder, and defects (pores and cracks) in the coating. With the same fraction of WC in the four WC–10Co–4Cr coatings, increasing the WC grain size, the mean free path of the binder is increased (Table 3). Based on the results obtained from an empirical formula [41], which relates the hardness of WC–Co cermet materials to the WC grain size and mean free path of the binder, the hardness decreases as the WC grain size increases. This is consistent with the nanohardness results of the WC–10Co–4Cr coatings in the present work. Moreover, this relationship can also be observed based on the corresponding indentation images. It has been reported previously that the hardness and Young’s modulus of WC (*H*_WC_ = 20–45 GPa and *E*_WC_ = 750 GPa) are higher than those of the binder (*H*_Co_ = 2–8 GPa and *E*_Co_ = 200 GPa) [43,44]. Thus, materials with the same indentation effect and harder WC particles showed higher hardness and Young’s modulus. The comparison of the indentation images of the N and M coatings (Figure 7) revealed that, when the WC particle size increased, the indentation area on the coating covered more binder and fewer WC particles. Therefore, the N coating showed a higher hardness and Young’s modulus than the M coating. 

The hardness and Young’s modulus are also related to the presence of the W_2_C phase in the coatings. The W_2_C phase exhibits higher hardness and a much lower Young’s modulus than the WC phase [13,45]. Thus, the highest content of W_2_C in the nanostructured N coating was one of the possible reasons for the highest hardness and the lowest Young’s modulus. In addition, the specific surface area of the nano-sized WC particle increases, which facilitates the dissolution of WC into the molten binder. In turn, the formation of brittle phases in the binder, such as Co_3_W_3_C, Co–Cr–W–C amorphous binder, was promoted, which caused the increase in hardness and decrease in the elastic properties of the binder [45], leading to higher hardness and lower Young’s modulus in the N coating than in other coatings. In contrast, the B1 coating containing both nano- and medium-sized WC particles, owing to the lower content of the W_2_C phase and dense structure, exhibited the highest Young’s modulus.

However, a different trend was observed in the cross-sections of the coatings. The lowest nanohardness and Young’s modulus were observed in the cross-sections of the N coating, which can be mainly attributed to the weak adhesion between the splats. Because the contact area between the splats in the cross-section is greater than that on the surface, its adhesion strength becomes a key factor affecting the mechanical properties of coatings in the cross-section direction. As the WC size increased, the bonding between the splats improved (Figure 4); thus, it exhibited an increasing trend for both the nanohardness and Young’s modulus in the cross-sections of the coatings. 

The large standard deviations of E and H indicated the heterogeneities of the microstructures and micromechanical properties of coatings, especially on the cross-sections, due to the lamellar structure. As the WC particle size increased, the mean free path of the binder was increased, resulting in a less uniform microstructure in each nanoindentation area (Figure 7). Therefore, this led to relatively larger standard deviations of E and H on the cross-sections of the M and B2 coatings (Table 5).

The Vickers microhardness values (*H*_V_) of the coating surfaces were measured to be compared with the nanoindentation test results and are shown in Table 5. The Vickers microhardness values were significantly lower than the nanohardness values for each sample. To understand the reasons for this, the differences between the characteristics of the two indentation tests were considered. The size of the indenters and the contact depth in the Vickers indentation test were much larger than those in the nanoindentation test. The Vickers indentation test is more affected by the overall defects and other characteristics of the coating, such as large pores, cracks, and weak cohesion between splats, than the nanoindentation test, consequently leading to lower hardness values. As discussed earlier, the porosity of the coating with bimodal-sized WC particles (B1 and B2 coatings) was lower than that with single-sized WC (the N and M coatings), leading to higher microhardness. As a result of the combined effects of the highest content of W_2_C and the highest porosity in the N coating, it showed the lowest microhardness. It should be noted that the determined hardness and Young’s modulus values also depend on the experimental conditions. For example, both indentation methods could be strongly affected by the testing load and time; thus, varied values can be obtained for the same sample. 

**Figure 9:**
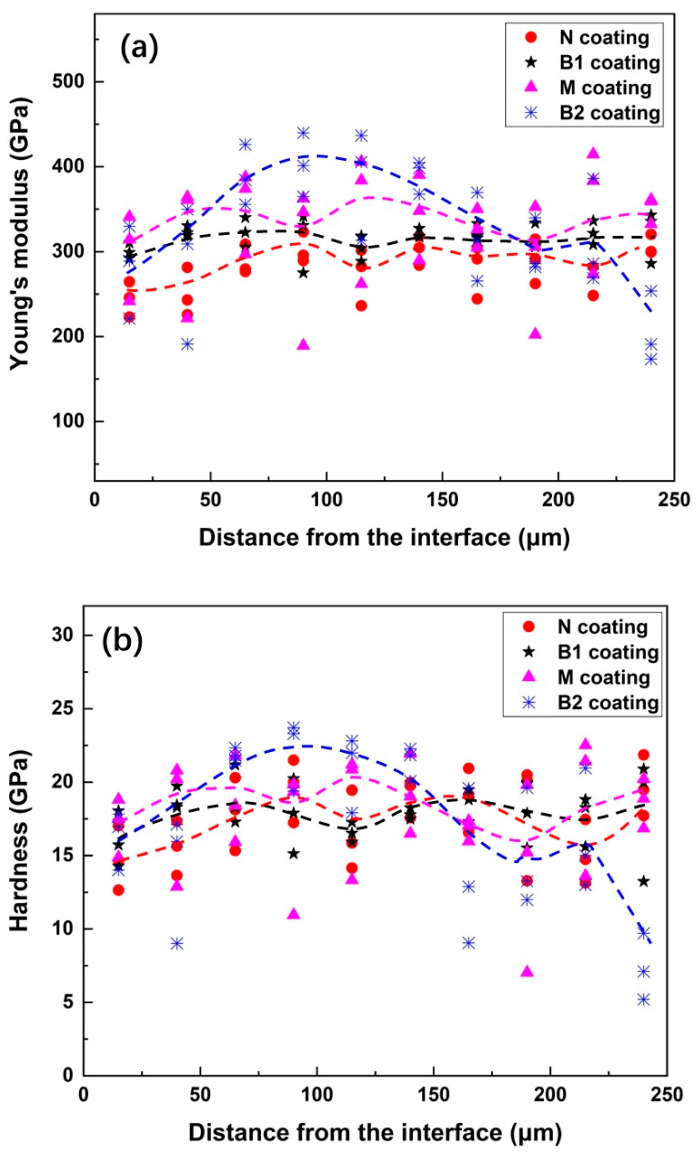
The through-thickness distributions of the Young’s modulus (E) and hardness (H) in the coatings. (**a**) The Young’s modulus; (**b**) the hardness.

Considering the deposition characteristics in the spraying process, the through-thickness distributions of E and H were investigated by nanoindentation to explore the consistency of the mechanical properties through the coating layer. The detected E and H values at each position along the coating are represented by symbols in Figure 9. The dashed line roughly describes the through-thickness distribution trend of E and H in each coating, which shows fluctuations along the coating. Among all four coatings, the B1 coating, with mixed nano- and medium-sized WC, presented relatively smooth E and H profiles along the coating thickness. Furthermore, the values tested at different points along the same distance from the coating–substrate interface are similar, with small dispersion. This reflects the uniformity of the micromechanical properties of the B1 coating. Conversely, the detected E and H values in the B2 coating containing medium-sized and coarse WC particles were the most dispersed, indicating the inhomogeneity of the micromechanical properties of the coating. Higher E and H values were observed in the middle part of the B2 coating (50–150 μm away from the coating–substrate interface). At the position near the interface and coating surface, its E and H decreased. This might be related to the uneven lamellar-like microstructures and complex stress states throughout the coating. However, it is usually desirable to use coatings with uniform properties for practical applications.

### 3.5. Fracture Toughness and Crack Path Observations

**Figure 10:**
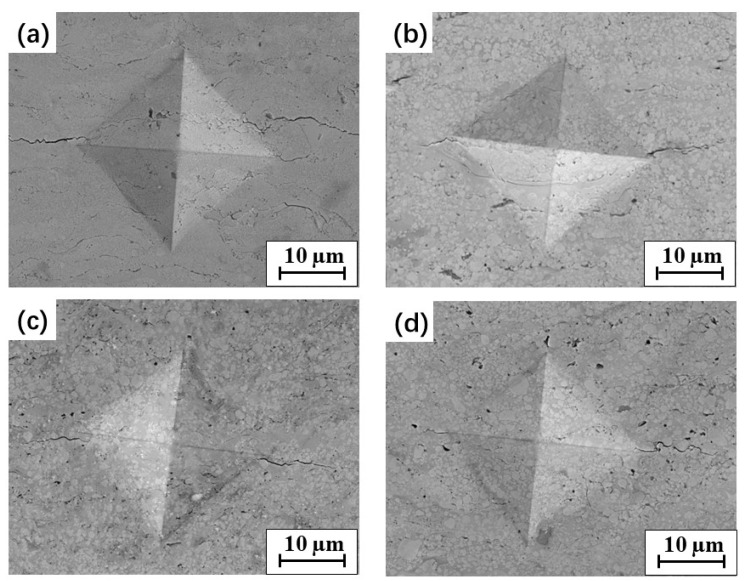
The typical SEM morphology of Vickers indentation imprints on the cross-sections of the studied WC–10Co–4Cr coatings: (**a**) N; (**b**) B1; (**c**) M; (**d**) B2.

Typical SEM morphologies of the Vickers indentation imprints on the cross-section of each WC–10Co–4Cr coating are shown in Figure 10. Indentation cracks were seen in the four types of coatings, emanating from the corners of the imprints and crack tips. The cracks mostly propagated parallel to the interface among spaying splats, and a few cracks were found along the direction perpendicular to the interface. The fracture toughness was calculated by Equation (1) based on the measurements of indentation imprint and crack length, as presented in Table 5. It is important to mention that the determined values of fracture toughness may not be accurate considering the anisotropy of crack propagation, but they should provide a useful means of assessing the differences among the specimens [46].

It was found that the fracture toughness of WC–10Co–4Cr coatings containing bimodal-sized WC particles was higher than that of those containing single-sized WC particles. The lowest fracture toughness was determined for the nanostructured N coatings, and as the WC particle size increased to the micron scale, the fracture toughness of the coatings obviously increased. It is well known that the fracture toughness of WC–10Co–4Cr materials is highly dependent on the binder and degree of oxidative decarburization [47]. As discussed earlier, with the increasing WC grain size, the mean free path in the binder was also increased and oxidative decarburization during spraying was reduced, thus limiting the generation of brittle phases with low toughness (such as W_2_C, Co_3_W_3_C, and Co–Cr–W–C amorphous binders). All of these factors are beneficial for increasing the fracture toughness of WC–10Co–4Cr coatings [17]. 

**Figure 11:**
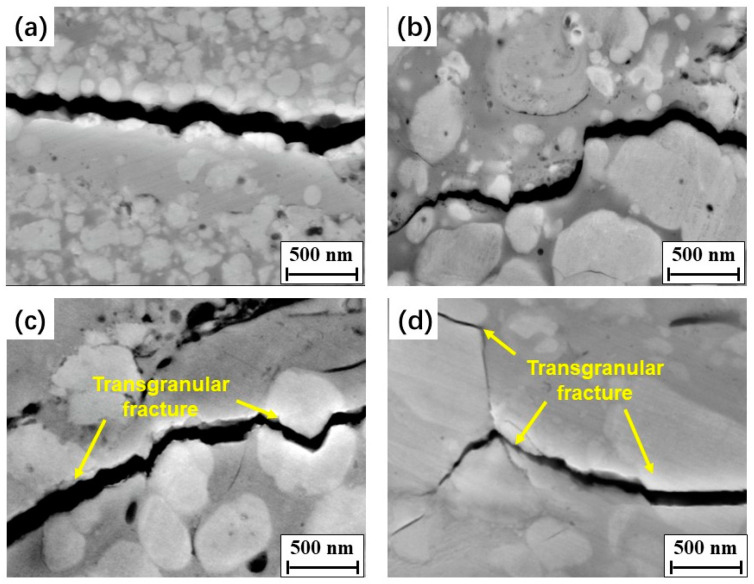
Typical indentation crack profiles in the studied WC–10Co–4Cr coatings: (**a**) N; (**b**) B1; (**c**) M; (**d**) B2.

Indentation crack paths were investigated to better understand the toughening mechanism, as shown in Figure 11. The intergranular fracture modes were the principal fracture modes in the coatings, presented as crack tips that propagated along the interface between the binder phase and WC particles. When the main crack met fine WC particles, such as nanoparticles in the N coating, it easily expanded along the boundaries between the binder and WC particles and a relatively smooth crack path in the N coating was observed (Figure 11a). As the WC grain size increased, crack deflection was seen when the crack met the coarse WC particles. Thus, more total energy was consumed during crack propagation, contributing to an increase in the fracture toughness. In the bimodal B1 coating, many nano-sized WC particles were uniformly distributed around the medium-sized WC particles, and crack propagation was slowed down owing to the obstruction of medium-sized WC particles, and the nanoparticles increased the phase interfaces (Figure 11b). All these factors caused an increase in the energy required for crack propagation, resulting in an increase in the fracture toughness of the B1 coating compared with the N and M coatings containing single-sized WC particles. As the WC size increased to medium size, transgranular fractures were observed in the M and B2 coatings, with the main cracks cutting through some of the medium and coarse WC particles, as shown in Figure 11c,d. The melting of the medium and coarse WC particles was limited in the M and B2 feedstock powders during thermal spraying. Thus, the impact energy of the solid WC particles on the substrate was higher than those of the other powders. This led to a large number of dislocations in coarse WC particles, as shown in Figure 6b, which can easily result in stress concentration [13]. When the main cracks extended to the coarse WC grains, the cracks easily nucleated and caused transgranular fractures in the WC grain. This can reduce the fracture surface, leading to a decrease in the total energy consumed during crack propagation. Moreover, the crack propagation and mechanical performance may be affected by the complex residual stress in the coatings. Therefore, it is desirable to further explore the residual stress in the coatings. 

### 3.6. Residual Stress

**Figure 12:**
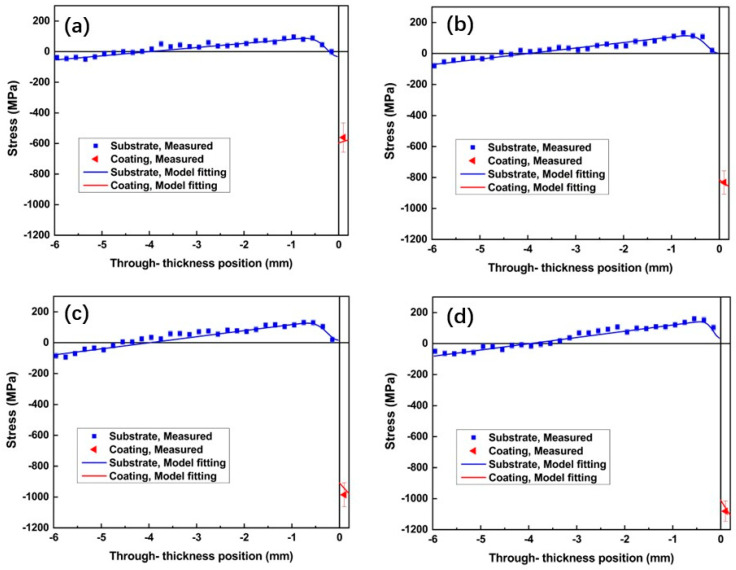
Stress distributions in the four WC–10Co–4Cr coating/steel substrate samples. Symbols with error bars represent experimental data, while lines correspond to an empirical model fitting the data sets. (**a**) N; (**b**) B1; (**c**) M; (**d**) B2.

The experimental stress measured in the steel substrate represents macro-stress, which refers to homogeneous stresses on a macroscopic scale [48,49], while, for the WC–10Co–4Cr coating, a composition of macro- and micro-stress is possible [49]. Figure 12 shows the through-thickness residual stress profiles in the WC–10Co–4Cr coating/steel substrate systems. Symbols with error bars represent experimental data points, whereas solid lines correspond to the model fitting of the data sets. 

Similar stress distribution trends were presented in the four coating/substrate samples with major features: compressive stress in the coatings (the moment of which is balanced by the bending of the substrate, causing linear stress distribution with a certain slope) and the presence of the 0.5-mm-thick compressive zone in the substrate adjacent to the interface. Such a stress state in the substrate and coating is mainly due to the several factors that generate stresses during spraying. They can be formulated within an empirical model of the progressive layer deposition model suggested by Tsui and Cline [50]. The details of the practical application of this model and stress parametrization for WC–10Co–4Cr coatings can be found in [49,51]. Within this empirical model, the following contributions are considered and can be evaluated from the experimental data:

(i) The main part is the large difference in thermal expansion coefficient (CTE) between the coating and substrate materials [49]. Since the CTE of the steel substrate (approximately 16 × 10^−6^/°C) is higher than that of the WC–10Co–4Cr coating (approximately 5 × 10^−6^/°C), the thermal–elastic mismatch between coating and substrate will cause mismatch strain, thus introducing residual compressive stress in the coating and tension in the substrate.
(ii) The deposition stress is characteristic of the spray process. It can be compressive, usually generated by dominated peening effects and typical for cold spray, or tensile, usually generated by dominated quenching effects and typical for thermal spray. For HVOF spraying, both effects are usually present [49] and the ultimate result of the sign of the stress is determined by the exact balance of the two competitive effects.
(iii) For the substrate, there is a typical feature in the near-to-interface region, a 0.5-mm-deep compressive zone that is associated with the peening effect [30,49]. There are two additional contributions at the near-to-interface region: (i) the peening effect by the high-speed droplets hitting the metal surface in the first moments of spraying, and (ii) the original stress in the substrate prior to the coating deposition, due to the grit blasting process on the substrate surface. Both are similar in action and usually generate a zone of compressive stress under the interface.

**Figure 13:**
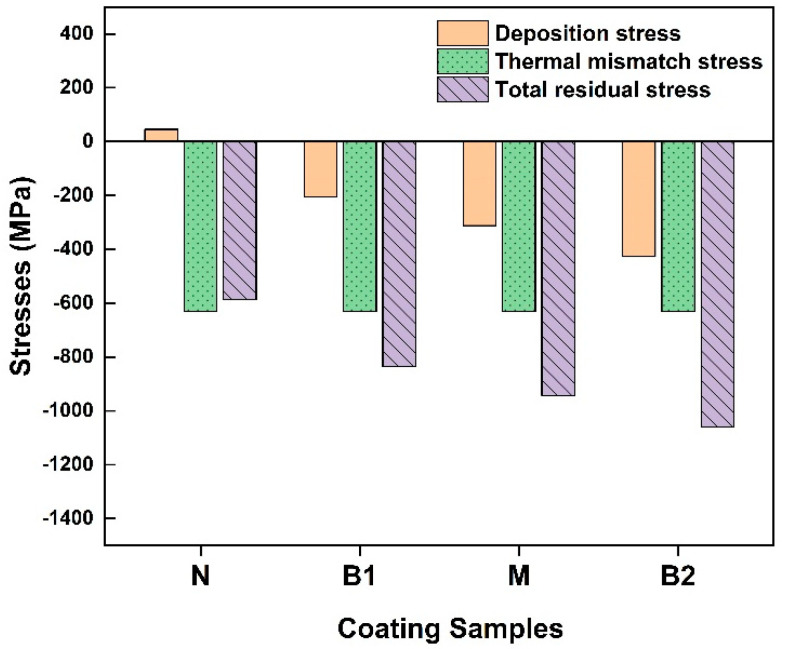
Decomposition of the total residual stress into the two components, the thermal mismatch stress and the deposition stress, for the coatings with different WC grain sizes.

The combination of the above contributions results in the stress distribution in the coating and substrate and can fit the experimental stress profile (Figure 12) while using only two parameters to characterize stress generated by spraying, the thermal mismatch and the deposition stress. The results of such decomposition are presented in Table 6 and illustrated in Figure 13.

The through-thickness macro-stress profiles of the coatings were estimated using the stress/momentum balance condition from the measured stress profiles in the substrates (indirect approach), as shown by the red solid lines in Figure 12. Although the coatings are relatively thin and stress variation in thin coatings is expected to be insignificant, the through-thickness gradient of compressive stresses distributions is visible and informative. The positive gradient in sample N is determined by the deposition stress (the thermal mismatch stress contribution has a negligible gradient) and in correlation with the positive sign of the deposition stress (Table 6). For the B1, M, and B2 samples, the deposition stress is negative thus there is a negative gradient of stress in the coatings for these samples. 

From the results shown in Table 6 and illustrated in Figure 13, the main formation mechanisms of residual stress in the WC–10Co–4Cr coating were revealed, quantified, and can be compared in terms of impact on the total acquired stress as follows [52]:

(i) The thermal–elastic mismatch stress produced between coating and substrate during the cooling process has the largest contribution, approximately −600 MPa for all samples. It is determined by the spraying conditions (temperature and CTE mismatch), which were close for all samples.
(ii) The deposition stress caused by the sudden solidification of the molten droplet during deposition, and the absolute values of compressive macro-stress in coatings, increase with increasing WC size. This might due to the more intense peening effect with coarse WC. As discussed above, the spread of droplets is more sufficient in the N coating than in the other coatings; this leads to a more remarkable quenching effect (solidification of the molten particle), which contributes tensile stress into the total balance, thereby leading to less compressive stress in the N coating.
(iii) The impact stress (or peening stress) in the substrate produced by high-speed particles impacted prior to the deposition of coatings. This is self-equilibrated stress existing in the substrate and does not impact the stress in coatings. The fact that this peening effect is more or less the same in all four samples suggests that it mainly comes from the grit blasting of the substrate surface and does not correlate with the powder used.

Many features are involved in the HVOF spraying process, such as a high temperature, large temperature change, high-speed impact, and layer-by-layer deposition. Considering the above complex mechanisms, after layer-by-layer deposition, it is bound to form uneven thermal expansion and splat shrinkage, resulting in through-thickness stress distribution in the substrate/coating system. Compressive macro-stresses were found in the four coatings, indicating the dominant effect of thermal–elastic mismatch between the coating and substrate materials on the residual stress of coating/substrate system.

In addition, the microstructure characteristics, such as the porosity of the coatings, also affect the total stress state in the coating. Thermal stresses in the N coating could be released more efficiently due to the maximum porosity compared to other coatings. All of these led to the lowest stresses in the N coating.

In addition to macro-stresses discussed above, micro-stresses are typical for composites and arise due to the different elastic, plastic, and/or thermal properties of the phases comprising the composites [49]. The compressive micro-stresses measured in the WC phase can be foreseen considering the CET mismatch between the WC particles and the binder. Due to the higher thermal expansion coefficient of the binder than that of the coating ($\alpha_{\mathrm{Co}}$ = 13.8 × 10^−6^/°C and $\alpha_{\mathrm{WC}}$ = 5.2 × 10^−6^/°C) [49], compressive stress is anticipated in the WC phase and tensile ones in the binder. However, the magnitude of the stresses largely depends on the volume fraction and can be very close to zero when the volume fraction is close to 100%, as for the WC phase in this composite. In fact, for the current samples, the experimental micro-stresses were evaluated with large uncertainty of 100–150 MPa to be sufficiently close to zero, and therefore negligible in comparison with the macro-stress component. Differently from the current case, micro-stresses were found significant and measurable in [49], though also with large uncertainty, because of the much larger volume fraction of the Co binder (>30%) in the samples sprayed by cold spray.

Thus, the only particle-size-dependent stress component is the deposition stress. Experimentally, as the size of WC particles increases, the absolute values of compression in the WC phase and overall coating are increased (Table 6 and Figure 13). Such phenomena can be related to the peening and contact behavior between the WC particles and binder. The kinetic energy of nano-sized solid WC particles in the N droplet under high impact pressure is lower than that of the other feedstock powders, leading to a reduced peening effect, thus limiting the compressive stress in the N coating. In fact, the peening effect in the N droplet containing nano-sized WC particles is so small that the overall deposition stress is tensile (Table 6) due to the overpowering effect of the quenching mechanism. The high porosity of the N coating indicates the weak cohesion between WC particles and binder and can further contribute to the effective stress relaxation at a low level of residual stress. 

In general consideration of composites, the macro-stress in the composite materials is the sum of the phased stress of each phase. The stress of the individual phase cannot perfectly represent the macro-stress state of the coating, due to the neglected effects of other phases. However, in the given composition of coating used in this study, the phase measurements of the WC phase (without investigating stress in the Co binder) are representative of the macro-stress of the WC–10Co–4Cr coating due to the high volume fraction of the WC phase. 

## 4. Conclusions

WC–10Co–4Cr feedstock powders containing different WC particle sizes were prepared using the new agglomeration–rapid sintering method, and the coatings were prepared using HVOF technology. The microstructures, phase compositions, and mechanical properties of the coatings were investigated. The through-thickness profiles of the WC–10Co–4Cr coatings and substrate materials were determined using neutron diffraction. The effect of the original WC size on the feedstock powders and coatings was discussed. The main conclusions of this study are as follows:

(1) The new agglomeration–rapid sintering method is beneficial for inhibiting decarburization and avoiding aggregation between the powder particles during feedstock powder preparation.
(2) Dense microstructures with less than 2% porosity were observed in all coatings. Owing to the large specific surface area, nano-sized WC is more prone to decarburization during spraying, resulting in a serious decline in the coating performance. The HVOF spraying process for nanostructured coatings should be strictly controlled to prevent serious decarburization.
(3) The decarburization was reduced and the mechanical properties were improved in the coatings as the WC size increased to micron scale. The bimodal coating containing both nano- and medium-sized WC particles exhibited optimal integrated mechanical properties (Young’s modulus, hardness, and fracture toughness).
(4) An anisotropic mechanical behavior was observed for each coating. Higher E and H values on the coating surfaces than in the cross-sections were measured, which were mainly affected by the bonding behavior between the splats in the coating.
(5) Fluctuant micromechanical properties were detected in the HOVF-sprayed coatings, especially in coatings containing coarse WC particles.
(6) Dominated by the thermal mismatch effect, compressive macro-stresses were developed in the coatings and compressive micro-stresses in the WC grains. As the WC particle size increased, the macro-stress in the coating considerably increased (in absolute value, remaining compressive).

## Figures and Tables

**Table 1:** Information on WC–10Co–4Cr spraying feedstock powders.

Specimen	Starting Powders, wt%				
	WC Particles			Co	Cr
	Nano (100–300 nm)	Medium (0.8~1.5 µm)	Coarse (4~5 µm)	1~2 µm	1~2 µm
N	86%	-	-	10%	4%
B1	25%	61%		10%	4%
M	-	86%	-	10%	4%
B2	-	61%	25%	10%	4%

**Table 2:** Spraying parameters of WC–10Co–4Cr by HVOF.

Parameter	Values
Kerosene gas flow rate (L/min)	0.37
Carrier gas (N_2_) flow rate (L/min)	0.4
O_2_ gas flow rate (L/min)	30.4
Powder feeding rate (g/min)	85
Spray distance (mm)	380

**Table 3:** Phase composition and microstructural parameters measured for the WC–10Co–4Cr coatings.

Coatings	WC (wt%)	W_2_C (wt%)	Co_3_W_3_C (wt%)	Mean Carbide Size (μm)	Mean Free Path (μm)	Porosity (%)
N coating	62.53	27.05	10.42	0.18	0.18	1.80
B1 coating	75.85	16.64	7.51	0.92	0.29	0.89
M coating	80.48	13.75	5.77	1.25	0.45	1.02
B2 coating	82.3	13.62	4.08	2.17	0.58	0.97

**Table 4:** EDS analysis results (wt%) of the detected points marked in Figure 6.

Point	Co	Cr	W	C
1	52.79	16.45	27.56	3.2
2	68.28	18.14	10.63	2.95
3	43.31	12.79	42.95	0.95
4	-	-	96.16	3.88
5	-	-	94.54	5.46

**Table 5:** Obtained mechanical properties of WC–10Co–4Cr coatings.

Coatings	Nanoindentation Test				Vickers Indentation	Fracture Toughness $K_{IC}$ (MPa·m^1/2^)
	Surface		Cross-Section			
	E (GPa)	H (GPa)	E (GPa)	H (GPa)	$H_{V}$ (GPa)	
N	324 ± 29	18.9 ± 2.4	295 ± 28	17.4 ± 2.7	12.2 ± 0.9	6.04 ± 0.76
B1	344 ± 22	18.5 ± 2.1	317 ± 20	17.8 ± 2.0	13.1 ± 0.8	7.88 ± 0.81
M	338 ± 23	18.3 ± 1.5	328 ± 37	17.7 ± 3.1	11.9 ± 0.8	7.19 ± 0.65
B2	341 ± 25	18.2 ± 1.9	335 ± 48	18.1 ± 4.3	12.9 ± 0.6	7.64 ± 0.85

**Table 6:** Decomposition of the average stress in coating into contributions (total stress = thermal mismatch stress + deposition stress).

Coating	Experimental Total Stress (Direct Method), MPa	Calculated Total Stress (Model, Indirect Method), MPa	Thermal Mismatch Stress (Model), MPa	Deposition Stress (Model), MPa
N	−561 ± 95	−587	−632	45
B1	−832 ± 74	−836	−632	−205
M	−985 ± 78	−943	−632	−312
B2	−1081 ± 67	−1059	−632	−427
